# Population Differentiation and Polygenic Selection in the Finnish Ayrshire Cows Selected on Genomic Information

**DOI:** 10.1111/jbg.70061

**Published:** 2026-07-04

**Authors:** Katri Sarviaho, Minna Koivula, Terhi Iso‐Touru, Pekka Uimari, Katja Hänninen

**Affiliations:** ^1^ Department of Agricultural Sciences University of Helsinki Helsinki Finland; ^2^ Natural Resources Institute Finland (Luke) Jokioinen Finland

**Keywords:** cattle, diversity, generation proxy selection mapping, genomic selection, selection signatures

## Abstract

The Finnish Ayrshire cattle belong to the Nordic Red breeds. The basis of selection in Nordic Red breeds shifted from traditional pedigree‐based breeding values to genomic breeding values between 2011 and 2014. Joint genetic evaluation and admixture among the Nordic Red breeds have led to the formation of a composite Nordic Red population; consequently, contemporary Finnish Ayrshire represents an admixed population. We identified recent selection signatures in the Finnish Ayrshire genome using two complementary approaches: the Hudson estimator of Wright's fixation index (F_ST_) and generation proxy selection mapping (GPSM). Hudson F_ST_ quantifies population‐differentiation between groups, whereas GPSM detects selection signatures within a single population by regressing birth year on SNP genotypes. The aim of this study was to identify temporal allele frequency changes in SNPs consistent with selection during the genomic era and to evaluate their associations with milk production and fertility traits in Finnish Ayrshire. Genotypes were available for 64,160 cows across 46,914 SNPs, and phenotypic data on milk production and fertility traits were available for 49,417 genotyped individuals. Based on Hudson F_ST_, 56 SNPs showed genetic differentiation between cows selected using pedigree‐based and genomic information. In addition, 54 SNPs exhibited temporal allele frequency changes consistent with selection according to GPSM. Overall, 11 SNPs were identified by both methods. Of the 54 SNPs, thirteen were associated with the interval from first to last insemination in Finnish Ayrshire heifers. These results suggest that a substantial proportion of SNPs exhibiting temporal allele frequency changes during genomic selection are associated with heifer fertility.

**Abbreviations**
Allele substitution effect: b
Average information restricted maximum likelihood: AI‐REML
Best linear unbiased prediction: (BLUP)
Breeding value: EBV
centiMorgan: cM
False discovery rate: FDR
Finnish Ayrshire: FAY
Generation proxy selection mapping: (GPSM)
Genome‐wide association study: (GWAS)
Genomic best linear unbiased prediction: (GBLUP)
Genomic breeding value: (GEBV)
Genomic relationship matrix: (GRM)
Hardy–Weinberg equilibrium: HWE
Hudson estimator of Wright's fixation index: Hudson F_ST_
Interval from first to last insemination: IFL
Linkage disequilibrium: (LD)
Minor allele frequency: (MAF)
Nordic Red dairy cattle: (RDC)
Nordic total merit index: (NTM)
Quantitative trait locus: (QTL)
Runs of homozygosity: (ROH)
Single nucleotide polymorphism: (SNP)
Site‐specific haplotype homozygosity between populations: Rsb
Wright's fixation index: F_ST_
Yield deviation for 305‐day fat yield: FY
Yield deviation for 305‐day milk yield: MY
Yield deviation for 305‐day protein yield: PY

## 1 Introduction

The Finnish Ayrshire (FAY) belongs to the Nordic Red dairy cattle (RDC). With 57,000 cows included in the national milk recording system, FAY represents the second most common dairy breed in Finland (ProAgria 2025). Since 2008, selection of FAY has been based on the Nordic total merit index (NTM), a joint evaluation by Finland, Denmark, and Sweden for the RDC breeds (NAV 2025a, 2025b). The highest weighted traits in the NTM are milk yield (weight factor of 1.02 for bulls and genomic tested cows, and 0.93 for cows without genomic testing but with own yield record), fertility (0.36), udder conformation (0.26), and udder health (0.26; NAV 2025b). Prior to 2011, breeding decisions were based on traditional breeding values (EBV) estimated using a pedigree‐based relationship matrix and best linear unbiased prediction (BLUP). In 2011, genomic information was incorporated into the estimation of breeding values for the primary traits in the NTM (NAV 2025a). This was followed by the inclusion of RDC females in the reference population for genomic breeding value (GEBV) estimation in 2014. As a result of joint genetic evaluations and the import of genetic material from other RDC subpopulations, the present‐day FAY population represents an admixed group.

Selection for dairy cattle traits is expected to change allele frequencies and increase genome‐wide autozygosity (Kim et al. 2013; Forutan et al. 2018; Tang et al. 2007), potentially driving population differentiation. To quantify such differentiation, Wright's fixation index (**F**_**ST**_; Weir and Cockerham 1984) is widely used to describe genetic divergence between selected lines or populations. Among its estimators, the Hudson estimator of F_ST_ (Hudson F_ST_) is commonly applied because it is insensitive to differences in sample size (Hudson et al. 1992; Bhatia et al. 2013) and is well suited for detecting selection signatures in admixed populations (Bhatia et al. 2013). However, F_ST_‐based approaches may have limited power to detect selection signatures associated with traits of complex genetic architecture (Rowan et al. 2021) and in populations with overlapping generations (e.g., Ochoa and Storey 2021). Therefore, F_ST_ should be complemented with other methods to effectively detect selection signatures.

One such method is generation proxy selection mapping (GPSM; Decker et al. 2012; Rowan et al. 2021), which was developed to detect allele frequency changes in single nucleotide polymorphisms (SNPs) across generations resulting from polygenic selection. GPSM identifies SNPs responding to artificial selection that does not leave distinct sweep‐like signatures in the genome (Decker et al. 2012; Grohmann et al. 2023). Briefly, GPSM fits a linear mixed model in which a proxy variable—for example, birth date—serves as the dependent variable, while the allele substitution effect, a random polygenic effect, and a residual term are included as explanatory variables (e.g., Decker et al. 2012). In practical terms, the resulting allele substitution effect indicates whether an allele has become more or less common in younger animals compared with older ones, thereby revealing consistent increases or decreases in allele frequency over time (Decker et al. 2012). GPSM is robust in distinguishing directional selection from random genetic drift (e.g., Decker et al. 2012; Rowan et al. 2021; Grohmann et al. 2023). In addition, GPSM can detect selection signals even in panmictic populations and over short time spans of less than ten years (Rowan et al. 2021). A genomic relationship matrix (GRM) is included to account for population structure, inbreeding, and non‐random sampling of genotyped individuals (Yang, Lee, et al. 2011; Rowan et al. 2021; Grohmann et al. 2023), thereby reducing spurious associations. GPSM has been successfully applied in beef cattle (Decker et al. 2012; Rowan et al. 2021, 2024), dual‐purpose cattle (Rowan et al. 2024), and pigs (Chen et al. 2022; Grohmann et al. 2023) to identify polygenic selection.

Despite selection promoting genetic improvement in desired traits, undesired haplotypes can be co‐inherited through genetic ‘hitchhiking’ caused by linkage disequilibrium (LD; Smith and Haigh 1974). Moreover, although polygenic selection induces only subtle allele frequency shifts, the cumulative effects of alleles can result in substantial phenotypic changes (Rowan et al. 2021). Consequently, large genomic regions contributing to inbreeding depression in certain traits may accumulate. However, both Hudson F_ST_ and GPSM capture genetic rather than phenotypic changes. The associations of identified variants—that show consistent changes in allele frequency over time—with certain traits may be assessed using a targeted association analysis, which fits a linear mixed model similar to that used in GPSM but with an observed phenotype as the dependent variable. A GRM is again incorporated to account for population structure, inbreeding, and non‐random sampling of genotyped individuals.

Identifying genetic variants under selection and linking them to phenotypic traits is essential for understanding the impact of genomic selection in livestock. However, the effects of genomic selection in FAY have been scarcely investigated, and polygenic selection remains largely unexplored. Furthermore, research on genetic differentiation within FAY is limited. Therefore, the aim of this study was to detect genetic differentiation based on Hudson F_ST_ between FAY individuals selected using traditional EBVs and those selected primarily using GEBVs. Additionally, we aimed to identify genetic variants showing temporal allele frequency changes consistent with selection for the first time in FAY using GPSM. Finally, we conducted an association analysis including the candidate SNPs identified using GPSM to explore the relationships of selected SNPs with milk production and fertility traits.

## 2 Material and Methods

In this study, RDC registered under the country code FIN (Finland) were considered FAY. Pedigree data were obtained from the Finnish Animal Breeding Association (Faba, Hollola, Finland) and included sire, dam, and birth date information for over 5.9 million FAY.

Genomic data were obtained from Nordic Cattle Genetic Evaluation (NAV, Aarhus, Denmark). The dataset comprised 64,160 genotyped FAY cows with information on 46,914 SNPs, born between 2009 and 2020 (Figure S1). Most animals were genotyped using the Illumina BovineLD v.1 BeadChip (Boichard et al. 2012), and low‐density genotypes had been imputed to medium density using FImpute software with default parameters (Sargolzaei et al. 2014) as a part of the routine genetic evaluation by NAV (A. Glasius, personal communication). Briefly, SNPs with a minor allele frequency (MAF) of at least 0.01 in RDC and samples with a call rate of at least 95% were accepted (Andreas Bøgh Poulsen, personal communication).

### 2.1 Genomic Data Quality Control

No missing genotype calls were present in the imputed dataset obtained from NAV. SNP positions were originally derived from the UMD3.1 assembly (A. Glasius, personal communication) and subsequently updated to the ARS‐UCD1.2 assembly coordinates (GCA_002263795.2; NCBI 2025) using the public version of the SNP map (M. Leino and G. Sahana, personal communication). As a result, 1080 SNPs located on the sex chromosomes or mitochondria, or identified as mis‐mapped under ARS‐UCD1.2, were excluded.

Quality control was performed using PLINK version 1.9 (Chang et al. 2015; Purcell and Chang 2021). Prior to analysis, SNPs with a minor allele frequency (MAF) ≤ 0.01 were removed to account for MAF within FAY and potential genotyping errors. In addition, SNPs deviating significantly from Hardy–Weinberg equilibrium (HWE; at *p* < 1 × 10^−6^) were excluded prior to the F_ST_ analysis. Following filtering, 42,110 SNPs remained for the Hudson F_ST_ analysis, and 43,641 SNPs were retained for construction of the GRM used in the GPSM and association analyses.

### 2.2 Hudson F_ST_

Hudson F_ST_ was used to assess genetic differentiation between FAY selected using EBVs and those selected using GEBVs. To account for the gradual introduction of genomic selection between 2011 and 2014 and to mitigate the effects of overlapping generations, animals were divided into two groups. The first group included cows born between 2009 and 2011 (5783 individuals), predominantly selected using BLUP. The second group included cows born between 2017 and 2020 (35,392 individuals), representing the most recent generation, given an average generation interval of 3.6 years (Sarviaho et al. 2023), and selected using GBLUP. Region‐specific genetic differentiation between the groups was estimated using Hudson F_ST_:

$$\text{Hudson}\;F_{\mathrm{ST}}=\frac{{\left({\tilde{p}}_{1}-{\tilde{p}}_{2}\right)}^{2}-\frac{{\tilde{p}}_{1}\left(1-{\tilde{p}}_{1}\right)}{n_{1}-1}-\frac{{\tilde{p}}_{2}\left(1-{\tilde{p}}_{2}\right)}{n_{2}-1}}{{\tilde{p}}_{1}\left(1-{\tilde{p}}_{2}\right)+{\tilde{p}}_{2}\left(1-{\tilde{p}}_{1}\right)},$$

where $n_{i}$ is the sample size (allele count) and ${\tilde{p}}_{i}$ is the sample allele frequency in group i (i ϵ {1, 2}) (Bhatia et al. 2013).

A sliding window approach was used to identify region‐specific genetic differentiation. Hudson F_ST_ values were averaged over 620 kb sliding windows with 200 kb steps along each chromosome. Windows with no SNPs and incomplete windows at chromosome ends were discarded. Given an SNP density of approximately 1 SNP/62 kb, this window size ensured approximately ten SNPs per window, while the step size, being well above the average SNP spacing, improved localisation of signals. For each window, the SNP with the highest Hudson F_ST_ was designated as the top tagging SNP. Next, the window‐level mean Hudson F_ST_ values were smoothed over three adjacent windows within each chromosome. Smoothed window means in the top 0.1% (per chromosome) were considered statistically significant.

To assess genome‐wide population differentiation, the numerator and denominator of the formula were averaged across loci, and the genome‐wide Hudson F_ST_ was calculated as the ratio of these averages (Bhatia et al. 2013). The standard error (**SE**) of the genome‐wide Hudson F_ST_ was estimated using a block‐jackknife approach, as recommended by Bhatia et al. (2013). Variance was computed by dividing SNPs into 4 Mb blocks—approximately corresponding to the extent of linkage disequilibrium of around 5 cM in cattle (e.g., Arias et al. 2009) – and recalculating the ratio of averages while leaving one block out at a time. The SE of the ratio of averages for genome‐wide Hudson F_ST_ was calculated as:

$${\mathrm{SE}}_{\mathrm{ROA}}=\sqrt{\frac{B-1}{B}\sum_{b=1}^{B}{\left(\theta_{{\mathrm{ROA}}_{\mathrm{loo},b}-}\theta_{\mathrm{ROA}}\right)}^{2}},$$

where B is the number of blocks (with > 1 SNP), $\theta_{{\mathrm{ROA}}_{\mathrm{loo},b}}$ is the ratio of averages with block b left out, and $\theta_{\mathrm{ROA}}$ is the ratio of averages across all loci.

Microsoft 365 Copilot (Microsoft 2026) was used to assist in generating the R code for the sliding window and block‐jackknife approaches.

### 2.3 Generation Proxy Selection Mapping

GPSM was used to identify SNPs associated with birth date, reflecting allele frequency changes over time. All genotyped cows born between 2009 and 2020 were included to capture the gradual introduction of genomic selection.

The GRM was constructed, and both GPSM and association analyses were performed using GCTA v1.94 (GCTA 2025). The GRM was constructed following the method of Yang, Lee, et al. (2011):

$$\frac{\sum_{i=1}^{n}\sum_{j=1}^{m}\sum_{k=1}^{m}\left(x_{\mathrm{ij}}-2p_{i}\right)*\left(x_{\mathrm{ik}}-2p_{i}\right)}{\sum_{i=1}^{n}2p_{i}\left(1-p_{i}\right)},$$

where p_i_ is the MAF, and x_ij_ and x_ik_ are the genotypes of individual j and k at locus i; n is the number of SNPs (43,641), and m is the number of individuals (64,160).

To construct a birth date proxy, the variable *AGE* was defined as the time from January 2009 to the individual's birth month, rounded down to full years. *AGE* ranged from 0 to 11 years, with a mean of 7.25 years (SD 2.87).

SNPs showing temporal allele frequency changes consistent with selection were identified by fitting the univariate genome‐wide linear mixed model:

$$y=\mu +x_{s}b_{\text{GPSM}}+\mathrm{Zg}+e,$$

where **y** is the vector of *AGE* values, μ is the mean *AGE*, **x**_**s**_ is the vector of SNP genotypes for each individual at candidate SNP s, b_GPSM_ is the allele substitution effect of SNP (s), **g** is the vector of random polygenic effects, and **e** is the vector of random residual effects (Yang, Manolio, et al. 2011; Rowan et al. 2021). **Z** is the incidence matrix relating the random polygenic effects to individuals. Random effects were assumed to be normally distributed with var.(**g**) = **G** $\sigma_{g}^{2}$ and var.(**e**) = **I** $\sigma_{e}^{2}$, where **G** is the GRM and **I** is an identity matrix. Variance components of the polygenic and residual effects ($\sigma_{g}^{2}$ and $\sigma_{e}^{2}$, respectively) were estimated using average information restricted maximum likelihood (AI‐REML). Following previous studies (Rowan et al. 2021, 2024; Grohmann et al. 2023), *p*‐values for b_GPSM_ were converted to *q*‐values using R package ‘qvalue’ (Storey et al. 2023). SNPs with *q*‐values below false discovery rate threshold (FDR < 0.1) were considered genome‐wide significant. Genomic regions surrounding GPSM‐significant SNPs were examined in the UCSC Genome Browser (using the ARS‐UCD1.2 assembly; Perez et al. 2025) to identify nearby genes; only genes with complete coding sequence annotations were retained.

### 2.4 Association of GPSM‐Significant SNPs on Milk Production and Fertility Traits

We explored the association of SNPs, with temporal allele frequency changes (GPSM‐significant SNPs) with fertility and milk production traits by a targeted association analysis. Phenotypic data on milk production and fertility traits were obtained from NAV. Yield deviations for production traits were derived from first‐lactation test‐day records collected between 1988 and 2023 using the joint Nordic random regression test‐day model (Lidauer et al. 2015). Yield deviations were available for 305‐day milk yield (MY, kg), 305‐day protein yield (PY, kg), and 305‐day fat yield (FY, kg). Fertility data consisted of the interval from first to last insemination in heifers (IFL0) and cows (IFL1), based on records from 1992 to 2024. These records were pre‐corrected for systematic environmental and permanent environmental effects using the joint Nordic fertility evaluation model (Muuttoranta et al. 2015). Thus, the yield deviations for production traits and the pre‐corrected fertility records represent the animal‐specific component of performance. For production traits, records were restricted to genotyped cows with at least three test‐day observations, whereas fertility records were available for all genotyped cows. In total, 49,417 genotyped cows had phenotypic information for at least one fertility trait (Table 1). The previously constructed GRM was also used in the association analysis. A linear mixed model was fitted:

$$y=\mu +x_{t}b_{\mathrm{AS}}+\mathrm{Zg}+e,$$

where **y** is the vector of phenotypic values (MY, FY, PY, IFL0, or IFL1), μ is the vector of phenotypic value for the trait, **x**_**t**_ is the vector of SNP genotypes for each individual at candidate SNP t, b_AS_ is the allele substitution effect of SNP t, **g** is the vector of random polygenic effects, and **e** is the vector of random residual effects (Yang, Manolio, et al. 2011). The **Z** matrix relates individuals to their polygenic effects. Random effects were assumed to be normally distributed with var.(**g**) = **G** $\sigma_{g}^{2}$, and var.(**e**) = **I** $\sigma_{e}^{2}$, where **G** is the GRM and **I** is an identity matrix. Variance components ($\sigma_{g}^{2}$ and $\sigma_{e}^{2}$) were estimated using AI‐REML. SNPs with *p*‐values below 0.0093 (Bonferroni‐corrected threshold: 0.05/54) were considered significant. Manhattan plots were created with R packages ‘qqman’ and ‘ggplot2’ (Wickham 2016; Turner 2023).

**TABLE 1:** Descriptive statistics for milk production and fertility traits. The records represent the animal‐specific component of performance after correction for systematic environmental and permanent environmental effects.

Trait	*n*	Minimum	Maximum	Mean	SD
MY (kg)	41,196	−3759.23	7188.51	2366.95	1062.25
PY (kg)	41,196	−133.24	236.55	85.20	36.75
FY (kg)	41,196	−243.19	367.06	89.37	57.81
IFL0 (days)	45,074	−111.07	204.18	−6.69	32.55
IFL1 (days)	46,116	−102.49	124.39	0.46	23.29

Abbreviations: *n* = number of individuals with records; SD = standard deviation.

## 3 Results

### 3.1 Genetic Differentiation During Genomic Selection Introduction (F_ST_ Method)

The genome‐wide Hudson F_ST_ across SNPs was 0.01 (SE 1.25 × 10^−4^). A total of 29 windows fell within the top 0.1% of the sliding‐window distribution. These windows encompassed a total of 333 SNPs and 29 tagging SNPs, indicating genetic differentiation between the two groups (Table S1). Across all SNPs, Hudson F_ST_ ranged from −5.03 × 10^−5^ to 0.60. Among the 29 tagging SNPs, Hudson F_ST_ values ranged from 0.04 to 0.60, with ten tagging SNPs having their Hudson F_ST_ values above 0.25.

### 3.2 SNPs Undergoing Temporal Allele Frequency Changes (GPSM Method)

The *p*‐values of the null SNPs (non‐significant; FDR ≥ 0.1) from the GPSM analysis followed the expected uniform distribution, whereas the *p*‐values of all SNPs deviated from uniformity (Figure S2). A total of 54 SNPs were associated with *AGE* (*q*‐value < 0.1) and were therefore considered to be under the effect of polygenic selection (Table S2); ten of these were also significant based on the Hudson F_ST_ analysis, with their F_ST_ values ranging between 0.27 and 0.60.

Allele substitution effects (b_GPSM_) of null SNPs on *AGE* were normally distributed with a mean close to zero, whereas the effects of GPSM‐significant SNPs (FDR < 0.1) were bimodally distributed, with peaks above and below zero (Figure S3). The allele substitution effects of significant SNPs ranged from −0.52 to 0.20 years (Table S2), with a mean of −0.11 years (SD = 0.16). The absolute effects ranged from 0.07 to 0.52 years, with a mean of 0.17 years (SD = 0.09). Overall, the b_GPSM_ values for 53 GPSM‐significant SNPs deviated by more than 3 SD from the mean (−0.11) of all SNPs. The most extreme effect (−0.52 years) was observed for rs3423093715 on chromosome 18, which was 2.59 SD from the mean of the significant b_GPSM_ values and 21.01 SD from the mean b_GPSM_ value of all SNPs.

### 3.3 Association of GPSM‐Significant SNPs on Milk Production and Fertility Traits

We performed an association analysis of the 54 GPSM‐significant SNPs. Associations with *p*‐value below the Bonferroni‐corrected threshold (< 0.05/54) were considered significant. Of the 54 SNPs identified as significant by the GPSM, thirteen were associated with the fertility trait IFL0 in heifers (Table 2, Figures 1 and S4), whereas no significant SNP associations were detected for MY, FY, PY, or IFL1 in cows (Figures S5–S8).

**TABLE 2:** Chromosomal position (chr) in base pairs (bp), minor allele frequency (MAF; in genotyped cows born between 2009 and 2020), allele substitution effect (b_AS_) and the corresponding standard error (SE), and *p*‐values for SNPs associated with IFL0 in the genome association analysis. Estimates of allele substitution effect (b_GPSM_) derived from generation proxy selection mapping are presented for this SNP set, while the complete results are provided in Table S2. Genes with transcription sites located within 1 Mb of the associated SNPs are listed; a full list of genes is provided in Table S3.

Chr	SNP rsID^1^	Position (bp)	MAF	b_AS_ (SE)	*p*‐value	b_GPSM_	Gene
1	rs42998005	30,620,433	0.48	0.87 (0.240)	3.014 × 10^−4^	−0.15	—
2	no‐rsID_10	33,135,887	0.28	1.05 (0.275)	1.318 × 10^−4^	−0.21	*FIGN*
2	no‐rsID_11	56,566,543	0.41	1.40 (0.256)	4.150 × 10^−8^	−0.20	*LRP1B*
2	no‐rsID_21	66,072,100	0.39	1.17 (0.252)	3.297 × 10^−5^	−0.16	*ACTR3*
6	no‐rsID_15	14,005,865	0.29	0.99 (0.269)	2.331 × 10^−4^	−0.18	*FAM241A*
7	no‐rsID_7	75,268,501	0.34	1.40 (0.267)	1.599 × 10^−7^	−0.25	*MAT2B*
18	rs3423093715	652,553	0.19	1.47 (0.282)	1.976 × 10^−7^	−0.52	*RPH3A*
17	no‐rsID_6	61,506,352	0.32	1.08 (0.269)	5.874 × 10^−5^	−0.25	*UQCRFS1*
19	no‐rsID_18	30,462,752	0.22	1.26 (0.298)	2.393 × 10^−5^	−0.18	*DNAH9*
19	rs43721195	40,410,495	0.42	−1.10 (0.232)	2.013 × 10^−6^	0.10	*MSL1*
21	no‐rsID_20	48,318,429	0.22	1.01 (0.290)	4.945 × 10^−4^	−0.17	*SSTR1*
23	rs41599851	34,614,057	0.21	1.00 (0.290)	5.432 × 10^−4^	−0.08	*PRP4*
24	rs42049116	31,132,914	0.30	1.41 (0.268)	1.385 × 10^−7^	−0.26	*ZNF521*

^1^ no‐rsID refers to a variant that does not have an assigned rsID on the ARS‐UCD1.2 assembly.

**FIGURE 1:**
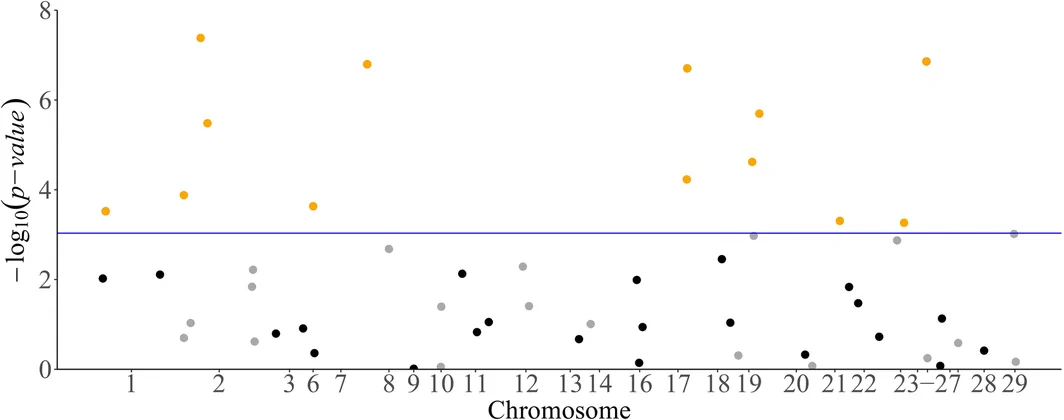
Manhattan plot of the association analysis for the interval from first to last insemination in heifers. The blue horizontal line indicates the statistical significance threshold of 0.0093 (Bonferroni corrected *p*‐value of 0.05/54), and significant SNPs are highlighted in orange. [Colour figure can be viewed at wileyonlinelibrary.com]

Allele substitution effects (b_AS_) of SNPs associated with IFL0 ranged from −1.10 days (SE 0.232) to 1.47 days (SE 0.282), with a mean of 1.00 day (Table 2). The most extreme effect was observed for rs3423093715, whose b_AS_ was 1.54 SD from the mean b_AS_ of all 54 SNPs tested. A positive b_AS_ indicates a longer interval from first to last insemination. Twelve of the IFL0‐associated SNPs showed positive allele substitution effects (minor allele as the reference allele) in the association analysis (b_AS_ = 0.87 to 1.47 days), but negative allele substitution effects in the GPSM (b_GPSM_ = −0.52 to −0.08 years). In contrast, the single IFL0‐associated SNP with a negative b_AS_ (−1.10 days) had a positive b_GPSM_ (0.10 years). Of the 13 SNPs associated with IFL0, nine were located within 1 Mb of the transcription start sites of 1689 genes in total (Table S3). These results suggest that the SNPs with temporal allele frequency changes during genomic selection are associated with fertility.

## 4 Discussion

Signatures of selection in cattle have been detected using complementary approaches that assess, for example, differences in the frequency of runs of homozygosity (ROH; e.g., Liu et al. 2021; Lozada‐Soto et al. 2022; Sarviaho et al. 2024) or site‐specific haplotype homozygosity (Rsb) between populations (e.g., Mastrangelo et al. 2020; Seo et al. 2022; Sarviaho et al. 2024). However, ROHs tend to arise in regions linked to Mendelian or otherwise simple traits, or near genes with large effects (Gorssen et al. 2021), whereas the Rsb statistic is mainly suited to identifying strong selective sweeps (Tang et al. 2007). Moreover, relatively few large‐effect quantitative trait loci (QTL) for milk production and fertility have been identified in the RDC (e.g., Kadri et al. 2014). Thus, in this study, GPSM—capable of detecting more subtle allele frequency shifts—was used to complement the F_ST_ analysis.

### 4.1 Pruning the Genomic Data

Following previous studies (e.g., Smaragdov et al. 2018; Rowan et al. 2021), genotype data were filtered for MAF ≤ 0.01 for GPSM, and for MAF ≤ 0.01 and deviations from HWE (*p* < 1 × 10^−6^) for the Hudson F_ST_ analyses. Thus, extremely rare variants, which may include genotyping errors, were excluded. To avoid over‐filtering, HWE filtering was applied at a relatively liberal threshold, and only for Hudson F_ST_ analyses. Genotype data used for the Hudson F_ST_ analysis were not pruned for LD, as LD pruning has been shown to have little effect on F_ST_ estimates in populations with multiple ancestries (e.g., Smaragdov and Kudinov 2020; Bercovich et al. 2025), such as the admixed FAY.

### 4.2 Genetic Differentiation During Genomic Selection

We used Hudson F_ST_ to assess population genetic differentiation during the introduction of genomic selection. In general, F_ST_ values < 0.05, 0.05–0.15, 0.15–0.25, and > 0.25 indicate little, moderate, great, and very great genetic differentiation, respectively (Wright 1978). For example, Hudson F_ST_ values between 0.002 and 0.015—indicating little genetic differentiation—have been reported between local Holstein herds (Smaragdov et al. 2018; Smaragdov and Kudinov 2020). In our study, the genome‐wide Hudson F_ST_ was similarly low (0.01), indicating limited differentiation between traditionally and genomic selected FAY cows. This outcome was expected because the FAY breeding programme underwent only modest changes during the gradual implementation of genomic selection (e.g., NAV 2025a). In addition, the average generation interval is 3.6 years in the FAY (Sarviaho et al. 2023); therefore, these results are consistent with a limited number of generations under genomic selection. Consequently, differentiation is expected to be local rather than genome wide.

Some Hudson F_ST_ estimates were negative, although close to zero. There is no consensus on how negative F_ST_ values should be interpreted: while Akey et al. (2002) consider negative values biologically meaningless, Smaragdov et al. (2018) suggest that they may reflect within‐population structure. Negative estimates may also arise from technical factors such as genotyping noise or imputation. However, because imputation accuracy from low‐density to medium‐density panels is considered high (Boichard et al. 2012), we consider the effect of imputation on our results to be negligible, given the quality control applied to genotype data. The unequal sample sizes between the two selection groups are also unlikely to bias our F_ST_ estimates because Hudson F_ST_ is robust to sample‐size differences (Bhatia et al. 2013). Finally, the sliding window approach employed here reduced statistical noise when identifying region‐specific differentiation. Overall, the identified regions with elevated F_ST_ likely reflect localised divergence between the two selection groups.

In total, 29 tagging SNPs indicated little to very great genetic differentiation (F_ST_ = 0.03–0.60) across all 29 chromosomes. Ten tagging SNPs showed Hudson F_ST_ values above 0.25 (0.27–0.60), indicating very great region‐specific genetic differentiation. Of the 29 SNPs tagging differentiated regions, three (no‐rsID_13, no‐rsID_9, and rs109885321 on chromosomes 3, 18 and 26, respectively) overlapped with selection signatures previously identified using ROH‐based methods in FAY (Sarviaho et al. 2024). These SNPs showed Hudson F_ST_ values of 0.30–0.34, indicating very great differentiation between the BLUP‐ and GBLUP‐selected groups.

### 4.3 SNPs Undergoing Temporal Allele Frequency Changes

GPSM is one of the few methods capable of detecting genomic changes resulting from polygenic selection in admixed populations such as FAY. GPSM fits a linear mixed model in which a temporal proxy—such as birth date—serves as the dependent variable, allowing the detection of temporal allele frequency changes over time. GPSM identifies SNP associations across the full MAF spectrum (Rowan et al. 2021)—although very rare variants were excluded in this study. Moreover, GPSM is designed to distinguish directional artificial selection from genetic drift (Decker et al. 2012; Rowan et al. 2021; Grohmann et al. 2023). Because the frequencies of selected alleles change consistently in direction over time, this results in an association of a SNP under the effect of selection with birth date, while drifting neutral variants will have approximately constant probability of a specific genotype (Decker et al. 2012), and thus, are not strongly predictive of birth date. However, this distinction may be influenced by factors such as sampling structure. The GRM accounts for relatedness, inbreeding, and overlapping generations, thereby reducing spurious associations of SNPs with both the proxy *AGE* in the GPSM and with the phenotype in the association analysis (e.g., Rowan et al. 2021; Grohmann et al. 2023). We applied GPSM to identify SNPs with temporal allele frequency changes in FAY cows during the era of genomic selection.

Although larger sample sizes increase the number of significant associations (e.g., Rowan et al. 2021; Grohmann et al. 2023), Bonferroni correction is widely considered overly conservative for genome‐wide analyses (e.g., Pearson and Manolio 2008). Following previous studies (e.g., Rowan et al. 2021, 2024; Grohmann et al. 2023), we therefore applied FDR control when assessing the significance of SNPs in GPSM. Of the 43,641 SNPs analysed, 54 SNPs were identified as significant (FDR < 0.1) with the GPSM in our study. Eleven SNPs that tagged differentiated regions based on the Hudson F_ST_ were also GPSM‐significant.

In GPSM, the allele substitution effect b reflects the responsiveness of SNPs to selection: the larger the absolute b_GPSM_ value for *AGE*, the greater the allele frequency change over time (e.g., Decker et al. 2012). SNPs with large b_GPSM_ values may be linked to large‐effect genes and may be less constrained by antagonistic pleiotropy, whereas SNPs with small absolute b_GPSM_ values may relate to smaller‐effect loci or loci with constraints due to antagonistic pleiotropy (Decker et al. 2012). Furthermore, Grohmann et al. (2023) suggest that smaller absolute b_GPSM_ values can arise when many traits are simultaneously selected for and when some traits have low heritability; under these conditions, genetic progress is slower and allele frequencies change more gradually, yielding smaller absolute b. Consistent with this, most b_GPSM_ values among GPSM‐significant SNPs were distributed around zero, with a mean absolute value of 0.17. This pattern may arise, for example, from the negative genetic correlation between milk production traits and fertility (e.g., Roxström et al. 2001; Kadarmideen et al. 2003).

Three GPSM‐significant SNPs (no‐rsID_13, no‐rsID_9, and rs109885321 on chromosomes 3, 18, and 26, respectively) overlapped selection signatures previously identified using ROH‐based methods in the FAY (Sarviaho et al. 2024) and all three also tagged divergence between the selection groups based on Hudson F_ST_. These three SNPs had moderate absolute b_GPSM_ values (0.19–0.23 years; Table S2), consistent with links to small‐effect loci and/or constraints from antagonistic pleiotropy. The SNPs on chromosomes 3 and 26 were in close proximity to regions previously associated with milk fatty‐acid composition (Palombo et al. 2018; Shi et al. 2019) and birth weight (Sugimoto et al. 2012) in Holstein.

Because individuals included in the analysis were born from 2009 onwards, the detected allele frequency changes primarily reflect dynamics during and after the introduction of genomic selection. However, the data set was biassed towards recent generations and included a small proportion of cows born in 2009–2010 that were selected with traditional pedigree‐based breeding values (Figure S1). This temporal imbalance increases sensitivity to recent allele frequency changes while reducing the detection of older selection signals (Rowan et al. 2021). Because the cows in our genomic data represent a group intensely selected for improved milk production, selection signatures related to milk production may have been masked from the GPSM in our study. While uneven temporal sampling has had negligible effects on overall results in previous GPSM studies (e.g., Rowan et al. 2021; Grohmann et al. 2023), contributions from earlier selection and sampling structure cannot be completely ruled out. Accordingly, the GPSM signals observed in this study likely reflect a combination of ongoing polygenic selection, the transition from pedigree‐based to genomic selection, sampling structure, and, to a lesser extent, genetic drift. Despite this, inclusion of these individuals provides a baseline for pre‐genomic selection, enabling partial characterisation of this transition.

### 4.4 Association of GPSM‐Significant SNPs on Milk Production and Fertility Traits

We performed an association analysis of the 54 SNPs identified by GPSM. Because the SNPs were preselected based on GPSM, the subsequent association analysis is enriched for variants undergoing temporal allele frequency changes and therefore reflects trait association conditional on ongoing selection rather than genome‐wide effects.

Despite its advantages, GPSM primarily detects subtle selection‐driven changes in allele frequencies. Therefore, GPSM is less suited to detecting selection in regions harbouring large‐effect QTL, where alleles can reach fixation rapidly (Rowan et al. 2021). However, relatively few large‐effect QTL for milk production and fertility have been identified in the RDC (e.g., Kadri et al. 2014). Moreover, the heritability of IFL is low (0.02–0.04; Muuttoranta et al. 2019) compared with that of milk production traits (0.33–0.44; Lidauer et al. 2015), and genomic selection is expected to particularly enhance selection for low‐heritability traits such as fertility (Meuwissen et al. 2001).

Because the desirable direction of IFL0 is a reduction in the interval, the results of the current study suggest that allele frequency changes are consistent with a decrease in variants associated with poorer fertility and an increase in variants associated with improved fertility during genomic selection. Associations were observed between GPSM significant SNPs and heifer fertility, but not cow fertility. This result was not unexpected, given that poor fertility is a major reason for culling in FAY heifers (ProAgria 2025). Therefore, cows that have achieved at least one parity are likely to exhibit less infertility than heifers (e.g., Heise et al. 2018). Furthermore, the genetic correlation between IFL0 and IFL1 is moderate (0.40; NAV 2025b), indicating that selection for IFL0 only partly affects the same genetic variants influencing IFL1. Finally, because genomic selection has had relatively limited time to act in FAY, realised selection is expected to be strongest in the most recent generations—primarily the heifers represented in our data. Indeed, average fertility indices exceeded the yield indices until 2022 (NAV 2026), supporting this interpretation.

We observed GPSM‐significant SNP associations for fertility traits, but none for milk production traits, which may reflect both biological and methodological factors. First, fertility traits have historically been difficult to improve using traditional pedigree‐based BLUP selection compared with milk production traits. As discussed above, older selection signatures may be masked in GPSM analyses, because of uneven temporal sampling (Rowan et al. 2021). While this may enhance the detection of SNPs undergoing recent changes in allele frequency, it might additionally obscure older selection signatures, like those related to milk production. Thus, the combination of GPSM and association analysis is expected to be enriched for variants undergoing recent selection, favouring traits currently under selection (e.g., fertility).

Several genes previously linked to fertility traits were located near the IFL0‐associated SNPs, supporting the relevance of the detected regions. However, given the two‐step design of the analysis, these findings should be interpreted as candidate signals, rather than a comprehensive representation of the genetic architecture of fertility. For example, no‐rsID_11, no‐rsID_6, and no‐rsID_18 were located near the transcription start sites of *LRP1B*, *RPH3A*, and *DNAH9*, respectively. *LRP1B* has been associated with somatic cell score in Holstein (Cole et al. 2011) and was overlapped by no‐rsID_11, which also tagged very great divergence between the two groups. Of the remaining IFL0‐associated SNPs, nine were located within 1 Mb of the start transcription sites of numerous genes (Table S3). These SNPs were closest to genes such as *UQCRFS1*, *ACTR3*, *MAT2B*, *ZNF521* and *SSTR1*. Notably, rs3423093715—the SNP with the third‐largest Hudson F_ST_ and the largest trait association effect on IFL0—was located near *UQCRFS1*, a candidate gene for early pregnancy and luteolysis in dairy cattle (Mezera et al. 2020). *ACTR3* has been associated with mastitis resistance in cattle (Zhong et al. 2023), *MAT2B* with age at first calving in Holstein (Tourchi et al. 2024), and *ZNF521* with fertility in RDC (Höglund et al. 2015). The SNP no‐rsID_7, located near *MAT2B*, showed very great divergence between the two selected groups. Similarly, no‐rsID_20 was associated with IFL0 and tagged very great differentiation between the two groups; this SNP was located near *SSTR1* and overlapped a QTL related to somatic cell score in FAY (Schulman et al. 2004). *SSTR1* is a candidate gene for growth and carcass traits in sheep (Zhao et al. 2018).

In summary, 29 SNPs tagged genetic differentiation between traditionally BLUP‐selected and GBLUP‐selected FAY based on Hudson F_ST_, and 54 SNPs showed temporal allele frequency consistent with directional genomic selection based on GPSM. In total, eleven SNPs both tagged region‐specific genetic differentiation and were GPSM‐significant. Thirteen GPSM‐significant SNPs were associated with interval from first to last insemination in heifers.

Overall, the results are consistent with polygenic selection affecting the FAY genome during the introduction of genomic selection, particularly in regions associated with fertility traits. However, it should be acknowledged that there are other valuable SNPs associated with traits relevant in the FAY breeding programme—not only the ones highlighted in the current study. Therefore, genome‐wide association studies (GWAS) using denser genomic data could provide complementary insight into the genetic architecture of milk production traits independent of temporal allele frequency changes. Functional validation of the identified regions would further clarify their biological roles.

## 5 Conclusions

We identified genetic differentiation at specific genomic variants between Finnish Ayrshire cattle selected using traditional pedigree‐based breeding values and genomic breeding values. The results indicate subtle but detectable allele frequency changes during the introduction of genomic selection. These changes are consistent with polygenic selection, particularly in regions associated with fertility traits.

Notably, variants showing temporal allele frequency shifts were associated with fertility traits in the context of ongoing selection. However, these findings reflect a combination of recent selection, transition to genomic selection, and sampling structure of genotyped individuals. Overall, the results provide insight into how selection has shaped genetic diversity in Finnish Ayrshire cattle and highlight candidate regions that may be relevant for future improvement of fertility in the era of genomic selection.

## Funding

Oiva Kuusisto foundation (personal grant “Suomi kasvaa ruoasta”; no. 20220102) has funded this research.

## Ethics Statement

The authors have nothing to report.

## Conflicts of Interest

The authors declare no conflicts of interest.

## Supporting information


**Figure S1:** Distribution of birth years among genotyped cows.
**Figure S2:** Quantile‐Quantile plot of expected and observed −log_10_(*p*‐value) for all genome‐wide SNPs (43,641; left) and null SNPs (43,587; right) in generation proxy selection mapping.
**Figure S3:** Distribution of allele substitution effects (*b*) for significant SNPs (*q* < 0.01; left) and null SNPs (*q* ≥ 0.1; right) in generation proxy selection mapping.
**Figure S4:** Quantile‐Quantile plot for association analysis for milk production traits (MY = milk yield (kg), PY = protein yield (kg), FY = fat yield (kg)) and fertility traits (IFL0 = interval from first to last insemination (days) in heifers; IFL1 = interval from first to last insemination (days) in cows). Associated SNPs (Bonferroni corrected *p*‐value < 0.05/54) are highlighted in orange.
**Figure S5:** Manhattan plot of the association analysis for milk yield. The blue horizontal line indicates the statistical significance threshold of 0.0093 (Bonferroni corrected *p*‐value of 0.05/54).
**Figure S6:** Manhattan plot of the association analysis for protein yield. The blue horizontal line indicates the statistical significance threshold of 0.0093 (Bonferroni corrected *p*‐value of 0.05/54).
**Figure S7:** Manhattan plot of the association analysis for fat yield. The blue horizontal line indicates the statistical significance threshold of 0.0093 (Bonferroni corrected *p*‐value of 0.05/54).
**Figure S8:** Manhattan plot of the association analysis for interval from first to last insemination if cows. The blue horizontal line indicates the statistical significance threshold of 0.0093 (Bonferroni corrected *p*‐value of 0.05/54).
**Table S1:** Genomic regions exhibiting significant differentiation (top 0.1% of smoothed window‐level mean Hudson F_ST_ values within chromosomes) between Finnish Ayrshire selected using pedigree‐based breeding values and the most recent generation selected using genomic information. SNPs located within selection signatures identified in Finnish Ayrshire (Sarviaho et al. 2024) are highlighted.
**Table S2:** Allele substitution effects (b_GPSM_) with corresponding standard errors (SE), *p*‐values, and associated *q*‐values for statistically significant SNPs in generation proxy selection mapping in the Finnish Ayrshire heifers and cows. SNPs located within selection signatures, identified in Finnish Ayrshire (Sarviaho et al. 2024) are highlighted.
**Table S3:** Genes with transcription sites located within 1 Mb of SNPs associated with IFL in Finnish Ayrshire heifers. The genes closest to each SNP are in bold.

## Data Availability

The data supporting the findings of this study are available from the Nordic Cattle Genetic Evaluation (NAV), Aarhus, Denmark, and the Finnish Animal Breeding Association (Faba), Hollola, Finland. Restrictions apply to the availability of these data, which were used under licence for this study. Data are available from the corresponding author upon reasonable request and with permission from NAV and Faba.
