# Supplementary material for: Population Differentiation and Polygenic Selection in the Finnish Ayrshire Cows Selected on Genomic Information
Source: J Anim Breed Genet. 2026 Jul 4;143(6):774–83. doi: 10.1111/jbg.70061 (PMC13639196; doi:10.1111/jbg.70061)
Supplement: Supplementary file 1 [file JBG-143-774-s001.docx]

**SUPPLEMENTARY MATERIAL**

Figure S1. Distribution of birth years among genotyped cows.

 Figure S2. Quantile-Quantile plot of expected and observed -log_10_(p-value) for all genome-wide SNPs (43,641; left) and null SNPs (43,587; right) in generation proxy selection mapping.

Figure S3. Distribution of allele substitution effects (*b*) for significant SNPs (q < 0.01; left) and null SNPs (q ≥ 0.1; right) in generation proxy selection mapping.

Figure S4. Quantile-Quantile plot for association analysis for milk production traits (MY= milk yield (kg), PY = protein yield (kg), FY = fat yield (kg)) and fertility traits (IFL0 = interval from first to last insemination (days) in heifers; IFL1 = interval from first to last insemination (days) in cows). Associated SNPs (Bonferroni corrected p-value < 0.05/54) are highlighted in orange.

Figure S5. Manhattan plot of the association analysis for milk yield. The blue horizontal line indicates the statistical significance threshold of 0.0093 (Bonferroni corrected p-value of 0.05/54).

Figure S6. Manhattan plot of the association analysis for protein yield. The blue horizontal line indicates the statistical significance threshold of 0.0093 (Bonferroni corrected p-value of 0.05/54).

Figure S7. Manhattan plot of the association analysis for fat yield. The blue horizontal line indicates the statistical significance threshold of 0.0093 (Bonferroni corrected p-value of 0.05/54).

Figure S8. Manhattan plot of the association analysis for interval from first to last insemination if cows. The blue horizontal line indicates the statistical significance threshold of 0.0093 (Bonferroni corrected p-value of 0.05/54).

Table S1. Genomic regions exhibiting significant differentiation (top 0.1 % of smoothed window-level mean Hudson F_ST_ values within chromosomes) between Finnish Ayrshire selected using pedigree-based breeding values and the most recent generation selected using genomic information. SNPs located within selection signatures identified in Finnish Ayrshire (Sarviaho et al., 2024) are highlighted.

| Chromosome | Start | End | Smoothed mean F_ST_ per region | Top SNP position (bp) | Top SNP F_ST_ |
| --- | --- | --- | --- | --- | --- |
| 1 | 137,776,231 | 138,276,230 | 0.03 | 138,184,605 | 0.06 |
| 2 | 56,312,109 | 56,812,108 | 0.05 | 56,566,543 | 0.53 |
| 3 | 67,243,576 | 67,743,575 | 0.03 | 67,472,539 | 0.3 |
| 4 | 50,036,528 | 50,536,527 | 0.02 | 50,239,474 | 0.07 |
| 5 | 29,521,868 | 30,021,867 | 0.03 | 29,820,580 | 0.07 |
| 6 | 13,788,606 | 14,288,605 | 0.03 | 14,005,865 | 0.35 |
| 7 | 74,986,109 | 75,486,108 | 0.05 | 75,268,501 | 0.59 |
| 8 | 13,968,381 | 14,468,380 | 0.02 | 14,464,214 | 0.05 |
| 9 | 26,064,186 | 26,564,185 | 0.04 | 26,353,699 | 0.05 |
| 10 | 37,744,738 | 38,244,737 | 0.04 | 37,977,888 | 0.04 |
| 11 | 28,759,352 | 29,259,351 | 0.04 | 29,138,659 | 0.34 |
| 12 | 47,227,903 | 47,727,902 | 0.06 | 47,465,304 | 0.6 |
| 13 | 43,060,596 | 43,560,595 | 0.02 | 43,362,383 | 0.04 |
| 14 | 279,949 | 779,948 | 0.03 | 490,055 | 0.05 |
| 15 | 1,609,024 | 2,109,023 | 0.02 | 1,896,472 | 0.04 |
| 16 | 46,887,279 | 47,387,278 | 0.03 | 47,094,623 | 0.06 |
| 17 | 29,333,170 | 29,833,169 | 0.02 | 29,582,177 | 0.05 |
| 18 | 652,553 | 1,152,552 | 0.08 | 652,553 | 0.57 |
| 19 | 30,214,615 | 30,714,614 | 0.03 | 30,462,752 | 0.27 |
| 20 | 31,721,756 | 32,221,755 | 0.03 | 32,009,781 | 0.07 |
| 21 | 48,161,002 | 48,661,001 | 0.04 | 48,318,429 | 0.3 |
| 22 | 33,978,967 | 34,478,966 | 0.02 | 34,222,916 | 0.08 |
| 23 | 11,748,759 | 12,248,758 | 0.03 | 11,991,841 | 0.05 |
| 24 | 60,791,651 | 61,291,650 | 0.01 | 61,058,160 | 0.04 |
| 25 | 40,109,981 | 40,609,980 | 0.02 | 40,282,487 | 0.05 |
| 26 | 19,930,090 | 20,430,089 | 0.04 | 20,196,747 | 0.3 |
| 27 | 42,339,701 | 42,839,700 | 0.02 | 42,638,160 | 0.07 |
| 28 | 29,402,771 | 29,902,770 | 0.02 | 29,654,854 | 0.06 |
| 29 | 26,426,723 | 26,926,722 | 0.04 | 26,854,265 | 0.05 |

Table S2. Allele substitution effects (b_GPSM_) with corresponding standard errors (SE), p-values, and associated q-values for statistically significant SNPs in generation proxy selection mapping in the Finnish Ayrshire heifers and cows. SNPs located within selection signatures, identified in Finnish Ayrshire (Sarviaho et al., 2024) are highlighted.

| Chr | SNP rsID^1^ | Position (bp) | MAF | b_GPSM_ | *SE* | p-value | q-value |
| --- | --- | --- | --- | --- | --- | --- | --- |
| 1 | no-rsID_26 | 26,728,419 | 0.08 | -0.15 | 0.020 | 5.26 × 10^-15^ | 8.83 × 10^-12^ |
| 1 | rs42998005 | 30,620,433 | 0.48 | -0.15 | 0.016 | 3.27 × 10^-20^ | 6.49 × 10^-17^ |
| 1 | rs41639109 | 106,271,610 | 0.38 | -0.08 | 0.016 | 5.96 × 10^-07^ | 6.67 × 10^-04^ |
| 2 | no-rsID_10 | 33,135,887 | 0.28 | -0.21 | 0.016 | 4.36 × 10^-37^ | 1.90 × 10^-33^ |
| 2 | rs43302650 | 33,302,435 | 0.25 | -0.08 | 0.022 | 8.17 × 10^-05^ | 6.86 × 10^-02^ |
| 2 | rs109395886 | 42,626,098 | 0.33 | 0.11 | 0.017 | 1.40 × 10^-10^ | 2.03 × 10^-07^ |
| 2 | no-rsID_11 | 56,566,543 | 0.41 | -0.20 | 0.017 | 1.12 × 10^-31^ | 4.43 × 10^-28^ |
| 2 | no-rsID_21 | 66,072,100 | 0.39 | -0.16 | 0.017 | 5.24 × 10^-21^ | 1.09 × 10^-17^ |
| 2 | rs109761133 | 128,074,493 | 0.17 | -0.49 | 0.017 | 2.61 × 10^-182^ | 5.70 × 10^-178^ |
| 2 | rs41637024 | 129,451,897 | 0.32 | -0.08 | 0.022 | 1.04 × 10^-04^ | 8.56 × 10^-02^ |
| 2 | rs109619673 | 131,672,626 | 0.10 | 0.16 | 0.037 | 1.95 × 10^-05^ | 1.77 × 10^-02^ |
| 3 | no-rsID_14 | 29,674,329 | 0.10 | -0.21 | 0.019 | 2.16 × 10^-30^ | 6.73 × 10^-27^ |
| 3 | no-rsID_13 | 67,472,539 | 0.20 | -0.20 | 0.017 | 4.79 × 10^-31^ | 1.61 × 10^-27^ |
| 6 | no-rsID_15 | 14,005,865 | 0.29 | -0.18 | 0.017 | 1.76 × 10^-27^ | 5.13 × 10^-24^ |
| 7 | rs109532189 | 1,803,756 | 0.38 | 0.19 | 0.018 | 6.09 × 10^-25^ | 1.56 × 10^-21^ |
| 7 | no-rsID_7 | 75,268,501 | 0.34 | -0.25 | 0.016 | 2.28 × 10^-53^ | 1.42 × 10^-49^ |
| 8 | no-rsID_4 | 30,219,965 | 0.35 | -0.29 | 0.016 | 1.33 × 10^-71^ | 1.45 × 10^-67^ |
| 9 | rs43593845 | 34,529,670 | 0.09 | 0.10 | 0.022 | 2.03 × 10^-06^ | 2.06 × 10^-03^ |
| 10 | rs109425380 | 37,713,144 | 0.23 | 0.11 | 0.027 | 4.17 × 10^-05^ | 3.64 × 10^-02^ |
| 10 | rs110231019 | 38,338,318 | 0.13 | -0.41 | 0.019 | 1.13 × 10^-105^ | 1.64 × 10^-101^ |
| 11 | no-rsID_19 | 29,138,659 | 0.24 | -0.17 | 0.017 | 7.43 × 10^-25^ | 1.71 × 10^-21^ |
| 11 | rs41665730 | 49,640,162 | 0.31 | -0.10 | 0.017 | 1.67 × 10^-09^ | 2.21 × 10^-06^ |
| 11 | no-rsID_37 | 66,074,422 | 0.11 | -0.10 | 0.020 | 4.83 × 10^-07^ | 5.70 × 10^-04^ |
| 12 | no-rsID_8 | 47,465,304 | 0.40 | -0.24 | 0.016 | 5.52 × 10^-50^ | 3.01 × 10^-46^ |
| 12 | rs41651029 | 56,021,401 | 0.29 | -0.12 | 0.017 | 5.16 × 10^-12^ | 8.04 × 10^-09^ |
| 13 | rs41602463 | 69,585,711 | 0.01 | -0.16 | 0.034 | 1.75 × 10^-06^ | 1.82 × 10^-03^ |
| 14 | rs108992893 | 16,203,731 | 0.02 | -0.16 | 0.035 | 7.48 × 10^-06^ | 7.10 × 10^-03^ |
| 16 | rs41640591 | 64,188,174 | 0.21 | -0.10 | 0.017 | 1.09 × 10^-09^ | 1.50 × 10^-06^ |
| 16 | rs110070881 | 67,460,891 | 0.32 | -0.12 | 0.023 | 4.74 × 10^-08^ | 5.75 × 10^-05^ |
| 16 | rs109115592 | 72,258,249 | 0.38 | 0.07 | 0.017 | 2.75 × 10^-05^ | 2.45 × 10^-02^ |
| 17 | no-rsID_6 | 61,506,352 | 0.32 | -0.25 | 0.016 | 6.98 × 10^-55^ | 5.08 × 10^-51^ |
| 18 | rs3423093715 | 652,553 | 0.19 | -0.52 | 0.016 | 4.21 × 10^-227^ | 1.84 × 10^-222^ |
| 18 | no-rsID_9 | 48,837,136 | 0.21 | -0.23 | 0.017 | 1.79 × 10^-42^ | 8.66 × 10^-39^ |
| 18 | rs42576394 | 60,583,784 | 0.36 | -0.19 | 0.016 | 1.95 × 10^-31^ | 7.08 × 10^-28^ |
| 19 | rs109463529 | 11,172,197 | 0.10 | -0.11 | 0.020 | 2.72 × 10^-08^ | 3.39 × 10^-05^ |
| 19 | no-rsID_18 | 30,462,752 | 0.22 | -0.18 | 0.017 | 6.75 × 10^-25^ | 1.64 × 10^-21^ |
| 19 | no-rsID_24 | 32,589,618 | 0.15 | -0.15 | 0.018 | 2.37 × 10^-17^ | 4.32 × 10^-14^ |
| 19 | rs43721195 | 40,410,495 | 0.42 | 0.10 | 0.016 | 2.45 × 10^-09^ | 3.15 × 10^-06^ |
| 20 | rs43238631 | 63,746,476 | 0.38 | 0.09 | 0.018 | 5.55 × 10^-07^ | 6.37 × 10^-04^ |
| 21 | rs42827294 | 9,865,536 | 0.48 | 0.08 | 0.017 | 2.21 × 10^-06^ | 2.20 × 10^-03^ |
| 21 | no-rsID_20 | 48,318,429 | 0.22 | -0.17 | 0.017 | 1.30 × 10^-24^ | 2.83 × 10^-21^ |
| 22 | no-rsID_23 | 12,834,708 | 0.11 | -0.16 | 0.019 | 2.14 × 10^-18^ | 4.06 × 10^-15^ |
| 22 | rs41611675 | 25,488,681 | 0.44 | 0.13 | 0.016 | 2.02 × 10^-15^ | 3.53 × 10^-12^ |
| 22 | no-rsID_27 | 54,732,366 | 0.16 | -0.14 | 0.017 | 7.62 × 10^-15^ | 1.23 × 10^-11^ |
| 23 | rs110641968 | 24,895,534 | 0.26 | -0.11 | 0.016 | 8.48 × 10^-12^ | 1.28 × 10^-08^ |
| 23 | rs41599851 | 34,614,057 | 0.21 | -0.08 | 0.017 | 1.41 × 10^-06^ | 1.50 × 10^-03^ |
| 24 | rs42049116 | 31,132,914 | 0.30 | -0.26 | 0.016 | 1.75 × 10^-56^ | 1.53 × 10^-52^ |
| 25 | rs111011688 | 1,405,987 | 0.21 | 0.10 | 0.022 | 7.49 × 10^-06^ | 7.10 × 10^-03^ |
| 26 | rs42089058 | 17,857,480 | 0.01 | 0.19 | 0.050 | 1.13 × 10^-04^ | 9.14 × 10^-02^ |
| 26 | rs109885321 | 20,196,747 | 0.22 | -0.19 | 0.017 | 3.08 × 10^-27^ | 8.39 × 10^-24^ |
| 27 | rs42124054 | 22,545,304 | 0.07 | 0.14 | 0.035 | 5.99 × 10^-05^ | 5.12 × 10^-02^ |
| 28 | rs42316001 | 36,110,502 | 0.41 | -0.10 | 0.017 | 1.10 × 10^-09^ | 1.50 × 10^-06^ |
| 29 | rs108940218 | 41,749,646 | 0.45 | -0.08 | 0.017 | 1.08 × 10^-06^ | 1.18 × 10^-03^ |
| 29 | rs110843280 | 44,090,714 | 0.19 | 0.10 | 0.022 | 9.11 × 10^-06^ | 8.46 × 10^-03^ |

^1^ no-rsID refers to a variant that does not have an assigned rsID on the ARS-UCD1.2 assembly.

Table S3. Genes with transcription sites located within 1Mb of SNPs associated with IFL in Finnish Ayrshire heifers. The genes closest to each SNP are in bold.

| Chr | SNP rsID^1^ | Position (bp) | Gene |
| --- | --- | --- | --- |
| 1 | rs42998005 | 30,620,433 | *-* |
| 2 | no-rsID_10 | 33,135,887 | ***FIGN***, *GCA*, *IFIH1*, *KCNH7*, *LOC107132260*, *LOC112442562* |
| 2 | no-rsID_11 | 56,566,543 | *LOC101908548*, ***LRP1B*** |
| 2 | no-rsID_21 | 66,072,100 | ***ACTR3***, *DPP10*, *GPR39*, *SLC35F5* |
| 6 | no-rsID_15 | 14,005,865 | *ALPK1*, *AP1AR*, *ENPEP*, ***FAM241A***, *LOC101906682*, *NEUROG2*, *PITX2*, *TIFA*, *ZGRF1* |
| 7 | no-rsID_7 | 75,268,501 | *CCNG1*, *HMMR*, ***MAT2B***, *NUDCD2* |
| 17 | no-rsID_6 | 61,506,352 | *ADAM1A*, *ALDH2*, *BICDL1*, *CFAP73*, *DDX54*, *DTX1*, *ERP29*, *GCN1*, *HECTD4*, *IQCD*, *LHX5*, *LOC112442047*, *LOC112442048*, *LOC112442049*, *MAPKAPK5*, *NAA25*, *OAS1X*, *OAS1Y*, *OAS1Z*, *OAS2*, *PLBD2*, *PTPN11*, *RAB35*, *RASAL1*, *RBM19*, *RITA1*, ***RPH3A***, *RPL6*, *SDS*, *SDSL*, *SLC8B1*, *TMEM116*, *TPCN1*, *TRAFD1* |
| 18 | rs3423093715 | 652,553 | *COG4*, *FUK*, *IL34*, *LOC100138269*, *LOC107133318*, *LOC112442247*, *LOC789753*, *MTSS1L*, *SF3B3*, ***UQCRFS1***, *VAC14*, *VSTM2B* |
| 19 | no-rsID_18 | 30,462,752 | *ADPRM*, *ARHGAP44*, ***DNAH9***, *ELAC2*, *LOC104975039*, *MAP2K4*, *MYH1*, *MYH2*, *MYH3*, *MYH4*, *MYOCD*, *PIRT*, *SCO1*, *SHISA6*, *TMEM220*, *ZNF18* |
| 19 | rs43721195 | 40,410,495 | *ARL5C*, *C19H17orf98*, *CACNB1*, *CASC3*, *CCR7*, *CDC6*, *CDK12*, *CSF3*, *ERBB2*, *FBXL20*, *FBXO47*, *GJD3*, *GRB7*, *GSDMA*, *GSDMB*, *IGFBP4*, *IKZF3*, *KRT10*, *KRT12*, *KRT20*, *KRT222*, *KRT23*, *KRT24*, *KRT25*, *KRT26*, *KRT27*, *KRT28*, *KRT39*, *KRT40*, *KRTAP1-1*, *KRTAP3-1*, *KRTAP3-3*, *KRTAP4-7*, *LASP1*, *LOC100141108*, *LOC100294937*, *LOC100295015*, *LOC100847371*, *LOC100848572*, *LOC100848612*, *LOC104968477*, *LOC540285*, *LOC618495*, *LOC618938*, *LOC777598*, *LOC787051*, *LOC787225*, *LOC788196*, *LOC788228*, *LOC788284*, *LRRC3C*, *MED1*, *MED24*, *MIEN1*, ***MSL1***, *NEUROD2*, *NR1D1*, *ORMDL3*, *PGAP3*, *PLXDC1*, *PNMT*, *PPP1R1B*, *PSMD3*, *RAPGEFL1*, *RARA*, *RPL19*, *RPL23*, *SMARCE1*, *STAC2*, *STARD3*, *TCAP*, *THRA*, *TNS4*, *TOP2A*, *WIPF2*, *ZPBP2* |
| 21 | no-rsID_20 | 48,318,429 | *CLEC14A*, *FBXO33*, *FOXA1*, *GEMIN2*, *LOC782598*, *MIA2*, *MIPOL1*, *PNN*, *SEC23A*, *SLC25A21*, ***SSTR1***, *TRAPPC6B*, *TTC6* |
| 23 | rs41599851 | 34,614,057 | *CSH2*, *LOC100298767*, *LOC788801*, *PRL*, *PRP-VII*, *PRP1*, *PRP14*, *PRP2*, *PRP3*, ***PRP4***, *PRP6*, *PRP8*, *PRP9* |
| 24 | rs42049116 | 31,132,914 | *KCTD1*, *PSMA8*, *SS18*, *TAF4B*, ***ZNF521*** |

^1^ no-rsID refers to a variant that does not have an assigned rsID on the ARS-UCD1.2 assembly.
